# Translationally Relevant Magnetic Resonance Imaging Markers in a Ferret Model of Closed Head Injury

**DOI:** 10.3389/fnins.2021.779533

**Published:** 2022-02-23

**Authors:** Elizabeth B. Hutchinson, Anakaren Romero-Lozano, Hannah R. Johnson, Andrew K. Knutsen, Asamoah Bosomtwi, Alexandru Korotcov, Anandakumar Shunmugavel, Sarah G. King, Susan C. Schwerin, Sharon L. Juliano, Bernard J. Dardzinski, Carlo Pierpaoli

**Affiliations:** ^1^Department of Biomedical Engineering, University of Arizona, Tucson, AZ, United States; ^2^Center for Neuroscience and Regenerative Medicine, Uniformed Services University of the Health Sciences, Bethesda, MD, United States; ^3^Department of Radiology, Uniformed Services University of the Health Sciences, Bethesda, MD, United States; ^4^National Institutes of Health, National Institute of Biomedical Imaging and Bioengineering, Bethesda, MD, United States; ^5^Department of Anatomy, Physiology and Genetics, Uniformed Services University of the Health Sciences, Bethesda, MD, United States

**Keywords:** ferret, traumatic brain injury, magnetic resonance imaging (MRI), translational modeling, meninges, brain stem

## Abstract

Pre-clinical models of traumatic brain injury (TBI) have been the primary experimental tool for understanding the potential mechanisms and cellular alterations that follow brain injury, but the human relevance and translational value of these models are often called into question. Efforts to better recapitulate injury biomechanics and the use of non-rodent species with neuroanatomical similarities to humans may address these concerns and promise to advance experimental studies toward clinical impact. In addition to improving translational aspects of animal models, it is also advantageous to establish pre-clinical outcomes that can be directly compared with the same outcomes in humans. Non-invasive imaging and particularly MRI is promising for this purpose given that MRI is a primary tool for clinical diagnosis and at the same time increasingly available at the pre-clinical level. The objective of this study was to identify which commonly used radiologic markers of TBI outcomes can be found also in a translationally relevant pre-clinical model of TBI. The ferret was selected as a human relevant species for this study with folded cortical geometry and relatively high white matter content and the closed head injury model of engineered rotation and acceleration (CHIMERA) TBI model was selected for biomechanical similarities to human injury. A comprehensive battery of MRI protocols based on common data elements (CDEs) for human TBI was collected longitudinally for the identification of MRI markers and voxelwise analysis of T2, contrast enhancement and diffusion tensor MRI values. The most prominent MRI findings were consistent with focal hemorrhage and edema in the brain stem region following high severity injury as well as vascular and meningeal injury evident by contrast enhancement. While conventional MRI outcomes were not highly conspicuous in less severe cases, quantitative voxelwise analysis indicated diffusivity and anisotropy alterations in the acute and chronic periods after TBI. The main conclusions of this study support the translational relevance of closed head TBI models in intermediate species and identify brain stem and meningeal vulnerability. Additionally, the MRI findings highlight a subset of CDEs with promise to bridge pre-clinical studies with human TBI outcomes.

## Introduction

Advancement in scientific knowledge and therapeutics for neurologic disorders is largely based on pre-clinical studies at the animal model level. Traumatic brain injury (TBI) research in particular has seen considerable progress over the past decade in the characterization of basic mechanisms, development of diagnostic markers and intervention strategies primarily driven by injury model experiments in rodents (Xiong et al., 2013; Zhang et al., 2014; Johnson et al., 2015; Chiu et al., 2016; Coleman and Höke, 2020). While rodent models are advantageous for reproducibly generating consistent and targeted TBI outcomes, their translational value is less certain and to date there has not been a successful migration of any therapeutic strategy for this disorder from rodent to human. At the center of these gaps is the challenge of faithfully recapitulating in a pre-clinical paradigm the same pathomechanisms that occur in humans. If this cannot be accomplished, then basic TBI research and therapeutic development is hindered or even misleading and so there is a need both to develop human-similar TBI models and to evaluate the translational relevance of experimental outcomes across these models.

Unlike brain disorders with clear genetic origin or that are linked to pathology of a specific cell type or neural circuit, TBI arises from direct biomechanical forces that affect brain tissue according to spatial location and mechanical tissue properties (Meaney and Smith, 2015). Consequently, there are a wide range of possible injury paradigms for modeling TBI and each may be drastically different from the others. While human TBI is also associated with multiple different etiologies and with heterogeneous pathologic outcomes, there are clear epidemiologic trends such as the high prevalence of mild TBI or concussion (Levin and Diaz-Arrastia, 2015; McKee and Daneshvar, 2015; Trifan et al., 2017) and selective injuries in vulnerable populations such as athletes (Giza et al., 2017; McCrory et al., 2017; McAllister et al., 2021) and military members (Warden, 2006; Hoge et al., 2008; Terrio et al., 2009; Kasper, 2015). It is then important to identify TBI paradigms that can be used to adequately study common or prioritized TBI types in humans. Notably, the most common TBI models – lateral fluid percussion and controlled cortical impact – are focal surgical models even though the majority of human TBI is closed-head and diffuse (Terrio et al., 2009; Bramlett and Dietrich, 2014; Faul and Coronado, 2015; Flanagan, 2015). While focal, penetrating models remain fundamental tools for TBI research there has also been considerable progress toward developing models based on biomechanical aspects of prevalent or population-specific human TBI, especially concussions and blast exposure.

A second approach to improving the translational relevance of pre-clinical TBI models is to consider experiments in non-rodent species, especially those with neuroanatomic features that are more similar to the human brain including swine, non-human primates, and ferrets (Manley et al., 2006; Morganti-Kossmann et al., 2010; Browne et al., 2011; Cullen et al., 2016; Schwerin et al., 2017, 2018, 2021; Vink, 2018). Because the biomechanical forces that give rise to TBI are dependent on the size, shape and composition of the medium through which they travel and act, brains that are larger, more gyrencephalic (folded) and contain greater relative white matter volume are assumed to provide a more translationally relevant basis for experimental TBI studies. Already, the investigation of blast and concussion in these species has been shown to have some human-similar outcomes including greater white matter involvement, vascular vulnerability, and clinical and behavioral outcomes that are parallel to TBI-related deficits in humans (Hutchinson et al., 2016, 2018; Schwerin et al., 2021).

In addition to improvement of the experimental TBI paradigm via species selection and injury biomechanics, the identification of outcome measures that can bridge experimental TBI models with human research and clinical diagnosis provides a powerful basis to associate pre-clinical findings with the human disorder. Among the few assessments that can be applied both for human TBI clinical diagnosis and as experimental outcomes in pre-clinical research, neuroimaging modalities – especially MRI scans – provide an important translational toolkit. In particular, a collection of radiologic markers for TBI have been identified as common data elements (CDEs) for human clinical diagnosis (Duhaime et al., 2010, 2012; Thompson et al., 2015) and the same modalities can be implemented also using pre-clinical MRI systems. The CDEs are systematically defined radiologic indications of different pathomechanisms organized in a four-tier system of: (1) conventional MRI (T1, T2, T2*, and diffusion weighted MRI) to detect structural changes and lesions, (2) diffusion tensor MRI (DTI) for advanced detection of microstructural alterations, (3) vascular MRI (perfusion weighted imaging, MR angiography and arterial spin labeling, ASL) and (4) MR spectroscopy to detect metabolic disruption. These MRI protocols have been standardized for widespread implementation and a common reporting system has been established to enable data standards for clinical research^1^.

In the present study, we evaluate a preclinical TBI model with high translational potential using CDE-based MRI tools to determine which radiologic markers are most evident following injury and characterize their anatomic localization and temporal course. A closed head injury model of engineered rotation and acceleration (CHIMERA) (Namjoshi et al., 2014; Whyte et al., 2019) was selected for its biomechanical similarity to high-incidence TBI types in humans – namely sports and automobile related head injuries. The CHIMERA model was applied in the ferret, which has a gyrencephalic cortex and relatively high white matter ratio as well as body dimensions that allow for *in vivo* MRI scanning using a 7T pre-clinical system. A translationally relevant MRI scan battery was collected at longitudinal time points before and following TBI, and registration and voxelwise analysis was performed to localize and follow structural, microstructural and vascular changes (i.e., CDE tiers 1–3). The most prominent MRI outcomes were brain stem involvement including T2, T2*, and diffusion changes, contrast enhancement of the meninges in a subset of injury paradigms and vascular injury including transiently reduced cerebral blood flow and blood-brain barrier (BBB) disruption. Taken together, this collection of radiologic findings indicated the subset of MRI outcomes that accompany the ferret CHIMERA model.

## Materials and Methods

### Ferret Cohort and Experimental Design

Eleven adult male ferrets were included in this study (Marshall Bioresources; North Rose, NY, United States) and were socially housed in pairs with continuous access to food and water and 12-h light/dark cycle. All animals were treated in accord with the guidelines of the Uniformed Services University of the Health Sciences and the National Institutes of Health’s *Guide for the Care and Use of Laboratory Animals* and all procedures were approved by the University Institutional Animal Care and Use Committee. Each ferret was imaged prior to injury and serially following TBI at specified time points of 3 h, 1 and 4 weeks following. Three of the ferrets were imaged additionally 6–19 weeks following the injury. A range of injury paradigms (Table 1) were induced from no applied injury (*n* = 2), to moderate device settings (*n* = 8, < 100 total psi), to severe device settings (*n* = 2, > 200 total psi). Both ferrets with the highest biomechanical load values reached endpoint criteria as predefined by our animal protocol (persistent motor deficits) at 4 and 7 days and so these were the final MRI time points collected for each. Another animal did not receive MRI scanning after the 3 h time point due to unexpected MRI scanner downtime.

**TABLE 1:** Individual data for each ferret in this study including the pressure (in pounds per square inch) input for the CHIMERA device and repetition paradigms (spaced at 5 min intervals) used for CHIMERA injury, the biomechanical loading score calculated from the injury paradigm and ferret age and weight on the day of injury.

Injury paradigm	Biomechanical loading score	Age on day of TBI (months)	Body weight on day of TBI (g)
No injury	0	26	1060
No injury	0	12	1770
1 × 60 psi	60	16	1260
4 × 15 psi	60	23	960
1 × 70 psi	70	13	1610
1 × 70 psi	70	8	1830
3 × 15 psi + 1 × 30 psi	75	6	1440
3 × 15 psi + 1 × 30 psi	75	6	1540
3 × 15 psi + 1 × 30 psi	75	6	1540
5 × 15–75 psi*	225	12	1755
4 × 60 psi*	240	16	1110

*indicates paradigms with severe motor deficit outcomes as described in the text.

### A Closed Head Model of Acceleration and Rotation in the Ferret

The CHIMERA device, which was originally described for the mouse to induce rotation/acceleration TBI (Namjoshi et al., 2014) has been scaled and adapted for the ferret body dimensions and anatomy (Whyte et al., 2019). All injuries in the present study were administered using a ferret CHIMERA device in a single session on the same day with variable input pressure (15–75 psi) and with one to four repetitions separated by 5-min intervals. A biomechanical loading parameter was calculated by the addition of psi values within the injury session (see Table 1). On the day of injury, the ferret was anesthetized using inhaled isoflurane (5% induction, 1–3% maintenance) and maintained on a heating pad. The ferret was fitted with a custom helmet-like interface over the head to avoid direct injury from the piston of the CHIMERA device to the skull and then placed onto the bed of the device and secured using the device straps. The settings of the device were controlled using the provided tablet interface and the piston was engaged digitally also using the tablet interface. Following injury, the ferret was monitored continuously as it recovered from anesthesia and for 3 h until it was transported to the MRI scan room for imaging.

### *In vivo* Magnetic Resonance Imaging Acquisition and Processing

A comprehensive MRI battery was developed and optimized for the ferret based on conventional and advanced MRI markers with translational relevance according to the CDEs for TBI radiology. All MRI scans were collected using a 7T Bruker MRI Biospec scanner with ParaVision 6.0 software and with a quadrature 86 mm RF coil. Ferrets were imaged under isoflurane anesthesia (5% induction and 1–3% maintenance) and held in a custom cradle system and head restraint using an MRI compatible head holder (Kopf Instruments; Tujunga, CA, United States) machined to fit a half tube with machined fittings to the Bruker sample positioning machinery. A circulating water pad was used to maintain the ferret body temperature during imaging and physiologic monitoring was performed for respiration, temperature, heart rate and blood O_2_ level. We note that isoflurane anesthesia was selected for advantages during long MRI scan sessions, but that this anesthesia may influence blood flow and T2*W outcomes (Tsurugizawa et al., 2016).

For contrast enhanced MRI, Gadoteridol (ProHance, Bracco, United States) was diluted in physiologic saline to 139.7 mg/mL (1:1) and approximately 5 mL volume of solution (equivalent to 0.3 mmol/kg or 1.5 human IV dose) was injected via a pre-placed i.p. butterfly catheter (23 gauge) without moving the animal position within the scanner.

The full MRI battery scan time was approximately 3-h and included weighted, scalar images and data for quantitative mapping as listed below. Given the importance of timing between TBI and MRI measurements made at the 3-h time point, the scan order within the battery was identical across all ferrets and time points.

#### Tier 1 Magnetic Resonance Imaging Protocols

• Two-dimensional multi-slice T2-weighted RARE images were collected with TE = 10/30/50/70/90/110 ms and TR = 5800 ms. For visualization of T2-weighted contrast, the images from all echoes were averaged. For T2 mapping, the R statistical package (version 4.1.0) and ANTsR package^2^ were used to read in the multi-echo data along with a binary mask generated manually for each brain volume and to perform fitting of the Carr-Purcell Meibloom Gill (CPMG) equation to determine T2 values at each voxel within the mask. The estimated magnitude image from this model was also output for each brain and used both for template generation and as a target for DTI processing.
• Three-dimensional T1-weighted MDEFT (T1W-MDEFT) MRI scans were acquired with TE/TR = 2/3000 ms and IR = 1100 ms. In addition to use as anatomical images, the T1W-MDEFT scans were also used to visualize and measure contrast enhancement by collection of a second image following i.p. administration of Gadoteridol (see tier 3).
• T2 -weighted fluid attenuated inversion recovery (FLAIR) was collected using a 2D RARE pulse sequence with TE = 22 ms, TR = 10 s, and IR = 2500 ms. This scan was also collected following contrast injection and directly visualized to identify potential meningeal enhancement (see tier 3).
• Three-dimensional T2* weighted MRI scans were collected using a multi-gradient echo pulse sequence with TE = 4/8/12/16/20 ms and TR = 40 ms were collected and the maps averaged across all echoes and visualized to determine the presence and conspicuity of blood products in the parenchyma expected to result from micro hemorrhages.

#### Tier 2 Magnetic Resonance Imaging Protocol: Diffusion Tensor Imaging

• Single-shot 2D echo planar imaging (EPI) with TE/TR = 55/6000 ms was collected in the forward and reverse phase encoding directions including four unweighted images and 14 diffusion weighted images with *b* = 800 s/mm^2^ and non-colinear directions. TORTOISE software was used for motion correction and blip-up/blip-down geometric distortion correction and also to fit the diffusion tensor and calculate metric maps including the fractional anisotropy (FA) and trace (TR) of the diffusion tensor (Irfanoglu et al., 2015, 2017).Single-shot 2D echo planar imaging (EPI) was collected in the forward and reverse phase encoding directions including three unweighted images, 30 images each for *b* = 800 and 1200 s/mm^2^. TORTOISE software was used for motion correction and blip-up/blip-down geometric distortion correction and also to fit the diffusion tensor and calculate metric maps including the fractional anisotropy (FA) and trace (TR) of the diffusion tensor.

#### Tier 3 Magnetic Resonance Imaging Protocols: Vascular Modalities

• Contrast Enhancement was evaluated by the injection of Gadoteridol and subsequent acquisition of post-contrast T1W-MDEFT and T2-FLAIR images. These were used for the generation of subtraction maps between the pre- and post-contrast MRI scans in order detect blood-brain barrier (BBB) disruption and injury of the larger vessels as well as meningeal enhancement.
• Two-dimensional single slice arterial spin labeling (ASL) was collected using an EPI pulse sequence with TE/TR = 17/5000 ms. Three different axial slice acquisitions were collected with locations selected consistently using the genu of the corpus callosum as a landmark reference for the most anterior slice and collection of additional slices 3 and 7 mm posterior to this reference. Maps for cerebral blood flow (CBF) were generated using custom Matlab code.

### Whole Study Image Registration

A T2-weighted anatomical template was generated using the ANTsR BuildTemplate command to register and combine the baseline images for each ferret (Avants et al., 2008). This template then served as the target for every ferret and timepoint and defined the template space for ROI analysis of T2 maps and contrast enhancement subtraction maps. Each T2-weighted anatomical was rigidly and diffeomorphically registered to the template and the transforms were saved and applied to the T2 maps to warp them from native to template space.

MDEFT and T2FLAIR contrast enhancement subtraction maps were similarly warped to the same space by first generating a template for each modality in the space of the T2-weighted template. This was accomplished using ANTs command line software to first rigidly registering each T1W-MDEFT and T2FLAIR volume to the native space T2 structural image of the same subject and scan session. The resulting transform was then applied along with the saved T2 affine, and diffeomorphic transforms to template space and the resulting volumes were averaged to generate T1W-MDEFT and T2FLAIR template volumes. These templates then served as targets for registration of individual T1W-MDEFT and T2FLAIR volumes from the full study and the resulting transforms were used to warp the same-space subtraction maps into the common template space.

The registration of DTI volumes was accomplished separately in order to take advantage of tensor-based registration techniques (Irfanoglu et al., 2016). All native space tensor images were used to generate a full-study DTI template and to simultaneously warp all DT volumes to this common space.

### Voxelwise Analysis

Template-space quantitative maps were concatenated and compared using FSL tools (Jenkinson et al., 2012). T2, DTI, and subtraction maps in template space were merged into a single 4D file using the fslmerge command and then randomize (Winkler et al., 2014) was used to generate T-statistic and p-statistic maps for a single group average *T*-test with covariate (biomechanical loading) at each time point (baseline, 3 h, 1 and 4–6 weeks). Threshold-free cluster enhancement was applied and contrast values of +1 and −1 were applied to the covariate term to evaluate positive and negative correlations of MRI metrics with biomechanical loading. The resulting p-stat maps were displayed as warm colors for positive correlation tests and cool colors for negative correlation tests and overlayed on average map volumes. Unless otherwise noted, the uncorrected *p*-value of less than *p* = 0.05 was used as a threshold.

### Template-Based Regions of Interest Analysis

In order to evaluate the longitudinal trajectory of T2, contrast enhancement and DTI metrics in specific regions of interest (ROIs) selected based on their prominence in the above voxelwise analysis, binary masks were generated by ITKsnap (Yushkevich et al., 2006) using the statistics maps and the semi-automated region growing or “snake” tool to segment voxels from brain stem, white matter and meninges with significant correlation with severity at different time points.

The resulting segmentation masks were then used to extract quantitative values from each brain volume and those values were plotted with respect to time after injury and visually coded by color according to TBI severity from orange (lowest biomechanical loading) to red (highest biomechanical loading). Data points from each individual ferret were connected by lines to visualize consistent longitudinal trajectories across animals in the study. For T2 and DTI metrics, each data point was normalized by dividing each data point by the baseline value for that ferret. For contrast enhancement subtraction maps, the data for each ferret was offset by subtracting the baseline value from each data point. Normalization and offsetting of the plot data were intended to aid in visualization of overall temporal trajectories. The R statistical software (version 4.1.0) with ANTsR (see text footnote 2, Avants et al., 2008) and ggplot (Wickham, 2009) packages was used for all steps of the template-based ROI analysis.

### Subject-Space Regions of Interest Analysis of Cerebral Blood Flow

Because of the limited number of slices, the ASL images were manually segmented using ITK-Snap v3.8.0 (Yushkevich et al., 2006) to create three whole-slice ROIs. The mean value of CBF was computed across slices.

## Results

**FIGURE 1:**
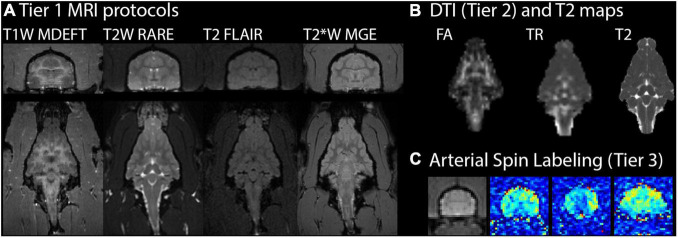
Ferret brain MRI battery images and maps. For one representative ferret in the study 19 weeks after mild injury, MRI scans with different contrasts (T1, T2, and T2*) are shown in the axial (top) and coronal (bottom) planes **(A)**. Quantitative MRI maps are shown in the coronal plane for fractional anisotropy (FA) and trace (TR) of the diffusion tensor and T2 **(B)**. Arterial spin labeling **(C)** maps for cerebral blood flow (pseudocolored) are shown at three different axial slice levels alongside an average value image (grayscale) of the middle slice for anatomic reference.

A comprehensive *in vivo* ferret MRI battery was established and applied in this study by adapting the human CDE MRI protocols and when possible quantitative maps were generated from the scans to enable voxelwise analysis. The scan parameters are given above and representative images and maps are shown in Figure 1 for T1W-MDEFT, T2-weighted multi-echo RARE with T2-mapping, T2-FLAIR, T2* MGE, DTI, and ASL. Contrast enhancement MRI was also successfully performed by repeating T1W-MDEFT and T2FLAIR scanning after administration of Gadoteridol.

### T1, T2, and T2* Magnetic Resonance Imaging Contrast After Traumatic Brain Injury

**FIGURE 2:**
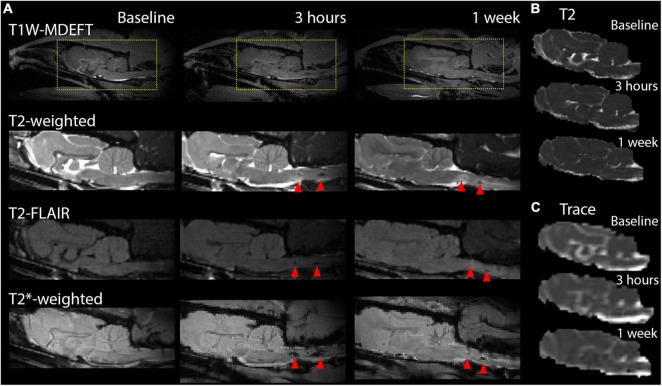
Markers of pathology on contrast weighted MRI scans, T2, and diffusion maps. Conventional MRI images for T1, T2, and T2* contrasts are shown at the sagittal midline prior to TBI and at 3 h and 1 week after to demonstrate the MRI profile of focal damage (red arrows) in this model including T2* hyperintensities post-TBI and T2-weighted hyperintensities at 1 week following severe injury. T2 **(B)** and DTI trace **(C)** maps are shown for the same brain and time points but do not feature strong focal abnormalities in the region of map coverage.

Magnetic resonance imaging scans of different contrast showed markers of hemorrhage and edema with a strong localization to the ventral brain, especially the brain stem and were prominent for the two severe cases in this study, but not conspicuous following less severe injury as shown in Figure 2. Prominent T2* hypointensities were the most evident marker from these images, especially at the 3 h time point. At later time points T2-weighted scans showed conspicuous regions of hyperintensity in a larger region surrounding the initial hemorrhage.

**FIGURE 3:**
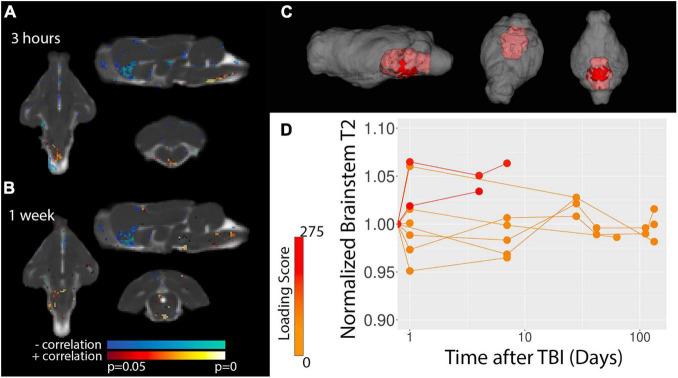
Voxelwise analysis of T2 values. Statistics maps are shown for the correlation between T2 and TBI severity for 3 h **(A)** and 1 week **(B)** after TBI. Results from baseline and 4–6 weeks did not show significant results. The template-space ROI mask for the brain stem (red, **C**) is shown with respect to the whole brain (gray) and template-based ROI values for the normalized T2 – i.e., divided by the baseline values – are plotted **(D)** with respect to time after injury where each data point represents a different scan session and the values for each ferret are connected by lines. Injury loading score color bar is shown for the values listed in Table 1.

To determine spatiotemporal patterns of T2 alterations, voxelwise analysis of correlation between T2 values and biomechanical loading was performed. Figure 3 shows voxels with the greatest significance were localized to the ventral medial brain, especially the brain stem and this effect was most prominent 3 h following TBI. Template-based ROI analysis of these regions (Figure 3B) showed that T2 was increased in the brain stem and confirmed the prominence of the finding for the most severe cases and earliest time point.

### Vascular and Meningeal Magnetic Resonance Imaging Outcomes After Traumatic Brain Injury

**FIGURE 4:**
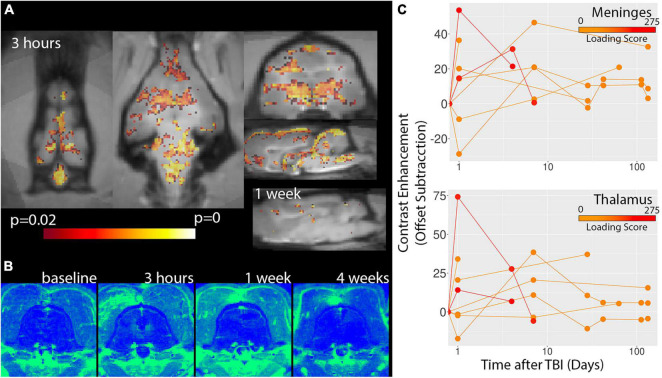
Voxelwise analysis of T1W-MDEFT contrast enhancement. *P*-value statistics maps for the correlation between T1W-MDEFT contrast enhancement and injury severity are shown for 3 h and 1 week time points **(A)** indicating extensive correlation for the early time point and very few voxels for the later. There were not significant clusters for the baseline or 4 week time points. Regions of Gadoteridol extravasation were visible on subtraction maps for severe cases of TBI **(B)** and most prominent for midline regions including meningeal spaces and deep structures. Template-based ROI analyses of the meninges and thalamus are shown for contrast enhancement **(C)** and indicate the greatest enhancement 3 h after injury for severe TBI. Injury loading score color bar is shown for the values listed in Table 1.

**FIGURE 5:**
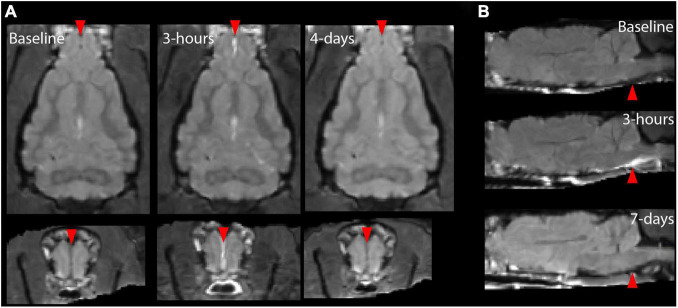
Individual T2 FLAIR post-contrast images showing regions of meningeal enhancement for two different ferrets 3 h after severe TBI. For the ferret shown in panel **(A)**, contrast enhancement was localized to the anterior midline region and for the ferret shown in panel **(B)**, contrast enhancement was found surrounding the brain stem. The red arrows indicate regions where extra cerebral enhancement was observable by eye for this modal on the day of injury but not 4–7 days after or at baseline.

The assessment of vascular injury by contrast enhancement on T2FLAIR and T1W-MDEFT MRI scans indicated regions of vessel damage and BBB disruption. Figure 4 shows voxelwise statistics maps and template-based ROI plots for T1W-MDEFT MRI scans with positive signal change following Gadoteridol administration. Multiple neuroanatomic regions were found to show severity dependent contrast enhancement including the thalamus and the brain stem. Significant voxels were also found for the dorsal midline region of the meningeal tissue. While statistical maps from the baseline and chronic stage maps did not show significant voxels, there were several clusters of significant voxels at the 1-week time point although it is unclear if these were contrast enhancement within the scan session or residual contrast agent from the previous (3 h) session. Contrast enhancement was visually prominent on the T2FLAIR MRI scans following Gadoteridol injection and especially in the midline meningeal regions either near the olfactory bulbs or the brain stem (Figure 5). Again, these were most prominent for the most severe cases.

**FIGURE 6:**
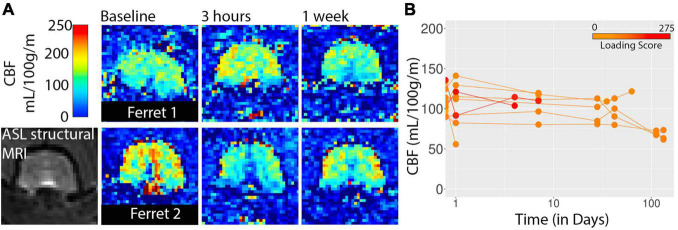
CBF maps from arterial spin labeling are shown for two ferrets **(A)** and whole brain values are plotted with respect to time after injury **(B)**. Injury loading score color bar is shown for the values listed in Table 1.

Cerebral blood flow was also assessed using whole brain ROI analysis for the three 2D slices acquired in this study using arterial spin labeling (Figure 6). There were no consistent indications of CBF alterations in the study although one of the most severe cases did exhibit a drop in global CBF at the 3 h time point and three low severity cases imaged at 16 and 19 weeks after injury did have lower CBF values than for earlier time points.

### Diffusion Tensor Magnetic Resonance Imaging Outcomes After Traumatic Brain Injury

**FIGURE 7:**
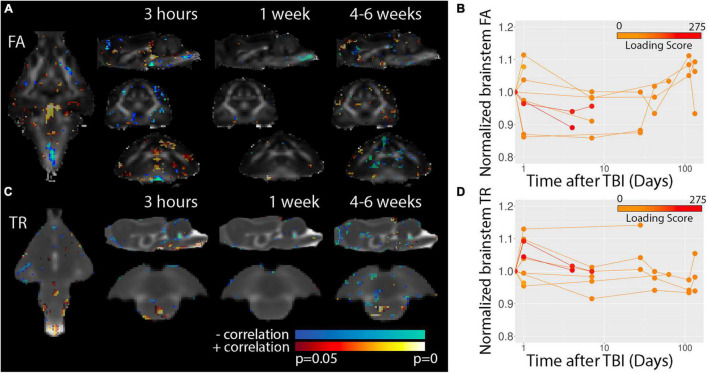
Voxelwise DTI analysis. *P*-value statistics maps for the correlation between FA **(A)** or TR **(C)** are overlayed on the average DTI maps for each of the timepoints indicated. Template-based analysis results for normalized brain stem FA **(B)** and TR **(C)** values are plotted with respect to time after injury for visualization of the temporal trajectory of DTI values in this region across all data collected. Injury loading score color bar is shown for the values listed in Table 1.

Diffusion tensor imaging was used to determine potential microstructural alterations following TBI in the ferret (Figure 7). Voxelwise maps for FA (Figure 7A) showed a somewhat disperse pattern of significant regions possibly indicating high levels of variability for this modality and metric. However, inspection of the brain stem region using template-based ROI analysis (Figure 7B) showed decreased FA at the 3 h time point across multiple ferrets and this decreased appeared to persist weeks after injury before returning to baseline values in the chronic period. Consistent with voxelwise results, DTI abnormalities were not evident for white matter (Supplementary Figure 1).

Voxelwise results for TR (Figures 7C,D) were indicative of a different spatiotemporal profile for post-TBI alteration than FA. Template-based ROIs in the brain stem showed early increases in TR for severe cases similar to findings for T2, but also showed increased TR for several milder cases. In addition, TR was found to be reduced in the brain stem in scans taken during the chronic period from less severe cases and regions of decreased TR were detectable by eye in the TR maps (Figure 2C) at 1 week but not earlier following severe injury.

## Discussion

This study provides a comprehensive assessment of radiologic outcomes following closed head rotation/acceleration TBI in ferrets. The major findings are the localization of post-CHIMERA injury to the brain stem region and prominence of subjectively detectable MRI outcomes – especially T2* hypointensities and T2 hyperintensities – in the severe cases, but little or no detectable subjective MRI outcomes in lower severity cases, although quantitative evidence from voxelwise analysis indicated T2 and DTI alterations across all severities. Contrast enhancement was evident within and beyond the brain stem including in meningeal spaces and deep midline structures and DTI outcomes were suggestive of persistent microstructural changes. Taken together these findings provide an MRI-focused basis for the development and evaluation of a translationally relevant animal model of TBI in the ferret.

### Involvement of the Brain Stem in the Ferret Closed Head Injury Model of Engineered Rotation and Acceleration Model

The most prominent imaging outcomes of this study were conspicuous abnormalities observable by conventional MRI contrasts (i.e., analogous to tier 1 CDE radiologic markers), especially T2*W hypointensities and T2W hyperintensities apparent as early as 3-h following TBI in the brain stem region and for the most severe cases. T2 hyperintensities are highly common outcomes for human TBI across different patient populations and TBI etiologies (Riedy et al., 2015) and have also been previously reported following surgical TBI in ferrets (Hutchinson et al., 2016, 2018) although with lower specificity to underlying cellular changes than other methods. The detection of microhemorrhage in this study by T2*W images is also relevant to the markers that accompany human TBI and have shown promise for the detection of traumatic axonal injury in milder cases including blast (Liu et al., 2016; Lotan et al., 2018). In the present study hemorrhages were localized to the brain stem and were not found diffusely in other areas of the brain, which suggests that the CHIMERA model using the settings and protocols of this study does not currently induce diffuse axonal injury with detectable microhemorrhages. However, the T2*W imaging protocols used in the present study may be advantageous for detection of such pathology in other more diffuse models. For example, ex-vivo studies of ferret brains following blast injury have found correspondence between T2*W hypointensities and histologic evidence of vascular injury (Schwerin et al., 2021).

Rodent studies employing the CHIMERA TBI model show preferential vulnerability of the ventral white matter tracts (Haber et al., 2017; McNamara et al., 2020) and in some cases more extensive diffuse white matter injury has also been observed (Namjoshi et al., 2016, 2017). In the present ferret CHIMERA experiments, ventral localization of injury was observed, but vulnerability of cerebral white matter tracts was not evident using neuroimaging (Supplementary Figure 1). This is somewhat surprising as we hypothesized vulnerability of the white matter to be consistent not only with rodent CHIMERA studies but also with the selective white matter damage found in human studies (Shenton et al., 2012; Hulkower et al., 2013). We expect that the head and brain biomechanics with the protocols and settings used in the present study preferentially affected the brain stem and cervical spinal cord and not the cerebral white matter. In the brain stem, hemorrhages, increased T2 was observed for both severe cases and T2 and DTI changes observed by template-based ROI analysis over the full sample set and time course. Clinically, the involvement of the brain stem is most prevalent in severe cases and has been associated with coma and fatality (Firsching et al., 2002; Kim, 2012; Rosenblum, 2015), which is not surprising given the basic functional roles of brain stem structures. At the same time, there is evidence that mild TBI and whiplash injury have overlapping pathomechanisms and outcomes (Ettlin et al., 1992; Beeckmans et al., 2017; Higgins et al., 2020) and that the brain may be affected in commonly occurring whiplash automobile accidents and sports injury. Differences in head and neck anatomy and brain geometry between ferrets and rodents most likely contribute to differences in neuroanatomic vulnerabilities to the CHIMERA procedure across species. While the study of injuries related to whiplash and brain stem injury may address key questions in TBI research, it is also possible to modify the protocols parameters of the ferret CHIMERA model according to other experimental objectives for example by changing the axis of rotation (Browne et al., 2011; Cullen et al., 2016), modified head kinematics based on biomechanical outcomes (Whyte et al., 2019) or modification of the device pressure settings or numbers of repetitions. Specifically, if the head and brain kinematics during injury by CHIMERA can be induced toward greater biomechanical strain in the cerebrum instead of the brain stem, then we expect that white matter may be selectively affected.

### Involvement of the Meninges and Vasculature

Contrast-enhanced imaging provided several key indications of vascular and meningeal damage in the ferret CHIMERA model. T2FLAIR hyperintensities in the meningeal spaces following the administration of Gadoteridol were observable by eye for both severe cases although localized in different regions including near the brain stem and on the midline of the anterior cortex. The presence of this marker was limited to the 3-h time point and not found in either brain 4 days after injury. This particular finding shows striking similarity to observations of post-contrast meningeal enhancement in humans imaged within 48 h of TBI (Turtzo et al., 2020). In this human study, meningeal enhancement was found in nearly half of those patients imaged and often in the absence of other CT or MRI outcomes. The meninges may play an important role in injury and recovery after TBI, but the pathophysiology of this system is not well understood. The observation of a translationally relevant MRI marker for post-traumatic meningeal damage in an animal model suitable to investigate basic biologic questions related to meningeal damage and function may provide an important translational tool for advancing TBI research.

In addition, the meningeal regions of contrast enhancement, quantitative mapping of T1W-MDEFT enhancement following contrast injection showed parenchymal regions within the brain that underwent extravasation of contrast agent consistent with damage to the blood brain barrier (BBB). In addition to the hemorrhagic brain stem regions identified by conventional modalities, additional regions of the brain showing enhancement included midline structures and deep bilateral regions such as the thalamus. There may have been more subtle effects present, but the variable uptake of contrast agent using intraperitoneal injections resulted in high variance for subtraction map values across scans. Future studies should consider using intravenous administration of contrast agent to reduce this variability and increase the sensitivity to smaller enhancement. The current study observations of enhancement were again most prominent for the most severe cases and the time course showed the greatest enhancement at 3-h followed by less at 4 days and return to baseline values by 1 week. The detection of contrast enhancement at 4 days could be influenced by decreased clearance of the contrast agent injected 3 days prior, however by generating subtraction maps between pre- and post- contrast images within the same scan session, we minimized this possibility. Unlike contrast enhancement findings, CBF was only decreased in a single ferret following TBI and ASL did not reveal widespread or consistent CBF alterations for other animals even those demonstrating meningeal enhancement and contrast agent extravasation in the parenchyma. This profile of vascular and meningeal injury bears resemblance to several of the key MRI outcomes in human TBI (Kenney et al., 2016; Griffin et al., 2019; Bolte et al., 2020) and provides multiple MRI modalities to assess different aspects of the cerebrovasculature in pre-clinical models.

### Diffusion Magnetic Resonance Imaging for Increased Sensitivity to Low Severity Outcomes and Persistent Abnormalities

Diffusion MRI techniques have been identified as promising for the detection of TBI pathology too subtle to be identified by more conventional approaches. The potential advantage DTI is that it can be used to probe tissue architecture and microstructure via metrics that report the magnitude and shape of water diffusion in tissue (Pierpaoli, 2002). In the present study diffusivity was increased in the brain stem acutely following TBI in both severe cases and in three of the mild cases, which is similar to findings in the cortex following controlled cortical impact in the ferret (Hutchinson et al., 2016) and may indicate acute vasogenic edema or other pathophysiology (Bramlett and Dietrich, 2004; Hutchinson et al., 2017). Unlike T2 values, diffusivity returned to baseline values days and weeks after injury suggesting a transient change in diffusivity despite persistent T2 elevation. It would be interesting to evaluate the physiologic underpinnings of this difference in future work.

Fractional anisotropy in particular has shown promise in both animal and human studies for following chronic neurodegeneration after TBI (Shenton et al., 2012; Hulkower et al., 2013). In the present study there was some evidence for an extended period of decreased brain stem FA beyond the acute period and for brains with less severe injury. However, the quality of diffusion MRI maps in this study and therefor the reproducibility across time-points was limited by short acquisition time and coarse resolution. We expect that expanded diffusion acquisition or improved DWI image quality would decrease image-related variability it falls to future studies with greater numbers of animals and perhaps with a greater portion of scan time dedicated to the collection of higher quality DTI scans to confirm and extend these initial DTI findings.

### Conclusion

A major objective for the advancement of TBI research is to increase the translational relevance of pre-clinical animal studies. In this work, we evaluated both a promising ferret model of closed head rotation/acceleration injury and MRI markers based on current clinical protocols. The animal model was found to incur injury of the brain stem with some involvement of the cerebral vasculature as well. This observation suggests that the current device and settings induce a whiplash-like injury, which may be directly relevant for studies of this injury mode or can serve as the basis for injury paradigm modification to target different TBI outcomes. The imaging markers identified following ferret CHIMERA were consistent with common human outcomes of T2*W hypointensities related to hemorrhage and T2 hyperintensities related to edema. Additionally, contrast enhancement of the meninges was observed with striking similarity to reports in humans and may provide a translational target for understanding meningeal injury after TBI.

## Data Availability Statement

The raw data supporting the conclusions of this article will be made available by the authors, without undue reservation.

## Ethics Statement

The animal study was reviewed and approved by Uniformed Services University of the Health Sciences Institutional Animal Care and Use Committee.

## Author Contributions

EH designed and conducted the experiments, processed and analyzed the data, and wrote the manuscript. AR-L and HJ processed and analyzed the data and wrote the manuscript. AKK processed and analyzed the data. AB collected *in vivo* MRI data. AK developed pulse sequences and processing protocols. AS collected *in vivo* MRI data and performed ferret CHIMERA experiments. SK assisted with ferret CHIMERA experiments and *in vivo* MRI scanning. SS provided expertise for ferret models and care. SJ provided expertise for ferret models and care. BD provided expertise for *in vivo* MRI methods. CP designed experiments, provided expertise for *in vivo* MRI, and developed processing and analysis methods. All authors contributed to the article and approved the submitted version.

## Conflict of Interest

The authors declare that the research was conducted in the absence of any commercial or financial relationships that could be construed as a potential conflict of interest.

## Publisher’s Note

All claims expressed in this article are solely those of the authors and do not necessarily represent those of their affiliated organizations, or those of the publisher, the editors and the reviewers. Any product that may be evaluated in this article, or claim that may be made by its manufacturer, is not guaranteed or endorsed by the publisher.

## Footnotes

1
http://www.commondataelements.ninds.nih.gov/

2
https://antsx.github.io/ANTsR

## Supplementary Material

Supplementary Figure 1 Template-based ROI analysis of T2 and FA showing negative findings for white matter alterations after CHIMERA in the ferret. Template-space ROIs are shown for the brain stem (red) from Figure 3 and white matter regions of the internal capsule (green) and cerebral WM (blue). T2 and FA values for each ferret are plotted across all time points for the white matter regions and no consistent alteration of these metrics was found in the WM. Injury loading score color bar is shown for the values listed in Table 1.
