# Supplementary figures and images for: Translationally Relevant Magnetic Resonance Imaging Markers in a Ferret Model of Closed Head Injury
Source: Front Neurosci. 2022 Feb 23;15:779533. doi: 10.3389/fnins.2021.779533 (PMC8904401; doi:10.3389/fnins.2021.779533)

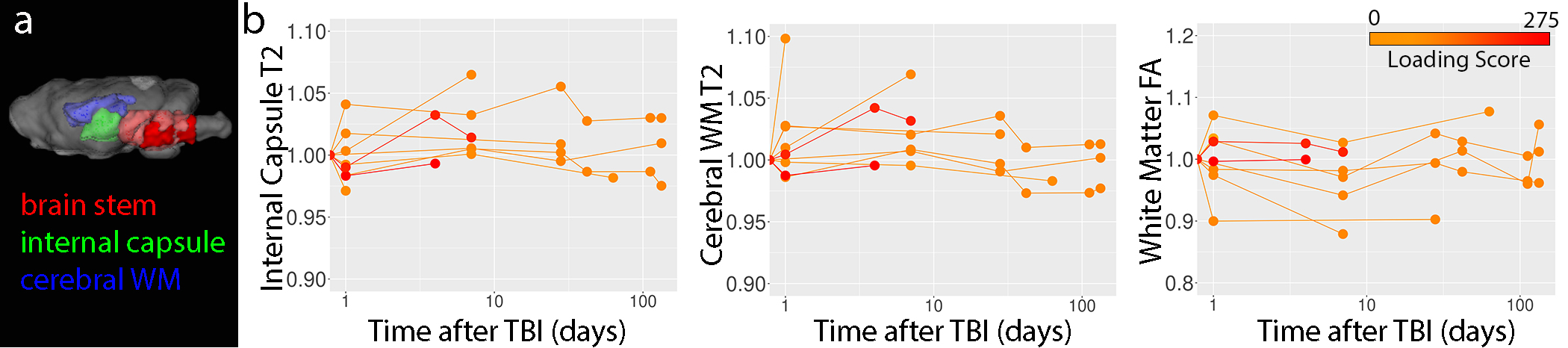

Supplement: Supplementary Figure 1 — Template-based ROI analysis of T2 and FA showing negative findings for white matter alterations after CHIMERA in the ferret. Template-space ROIs are shown for the brain stem (red) from Figure 3 and white matter regions of the internal capsule (green) and cerebral WM (blue). T2 and FA values for each ferret are plotted across all time points for the white matter regions and no consistent alteration of these metrics was found in the WM. Injury loading score color bar is shown for the values listed in Table 1. [file Image_1.jpg]
